# Feasibility of surface guided radiotherapy for patient positioning in breast radiotherapy versus conventional tattoo-based setups- a systematic review

**DOI:** 10.1016/j.tipsro.2022.03.001

**Published:** 2022-04-16

**Authors:** Wesley Naidoo, Michelle Leech

**Affiliations:** Applied Radiation Therapy Trinity, Discipline of Radiation Therapy, Trinity St. James’s Cancer Institute, Trinity College, Dublin, Ireland

**Keywords:** Surface guided radiotherapy, Breast cancer, Setup accuracy, Tattooless, Tattoos

## Abstract

**Background:**

Traditionally tattoos are used for patient setup in radiotherapy. However they may pose challenges for the radiotherapists to achieve precise patient alignment, and serve as a permanent visual reminder of the patient’s diagnosis and often challenging cancer journey. The psychological impact of tattoos has been recognized in recent years. The increasing complexity of treatment techniques and the utilization of hypofractionated regimes, requires an enhanced level of accuracy and safety. Surface guided radiotherapy (SGRT) enables improvements in the accuracy and reproducibility of patient isocentric and postural alignment, enhanced efficiency, and safety in breast radiotherapy.

**Purpose:**

The aim of this review was to compare the accuracy and reproducibility of SGRT to conventional tattoo-based setups in free-breathing breast radiotherapy and to determine if SGRT can reduce the frequency of routine image guided radiotherapy (IGRT).

**Materials and Methods:**

A systematic literature review was performed as per PRISMA guidelines. Papers identified through PubMed, Embase, Web of Science and Google Scholar database searches between 2010 and 2021, were critically appraised. Systematic, random, mean residual errors and 3D vector shifts as determined by IGRT verification were analysed.

**Results:**

A review of 13 full papers suggests SGRT improves the accuracy and reproducibility of patient setup in breast radiotherapy with consistent reductions in the residual errors. There appears to be a good correlation between SGRT setups and radiographic imaging. The frequency of IGRT and the corresponding dose could potentially be reduced. Additionally, SGRT improves treatment efficiency.

**Conclusion:**

SGRT appears to have improved the accuracy and reproducibility of patient setup and treatment efficiency of breast radiotherapy compared to conventional tattoo/laser-based method, with the potential to reduce the frequency of routine IGRT. The reliance on tattoos in breast radiotherapy are likely to become obsolete with positive implications for both patients and clinical practice.

## Introduction

### Breast cancer and radiotherapy

Breast cancer is the most common form of cancer in the UK with approximately 55,000 women diagnosed each year. The 5-year and 10-year survival rates are 86% and 78% respectively, which can be attributed to improvements in screening, diagnosis, and treatment [1]. Adjuvant radiotherapy is commonly administered to the chestwall, whole breast or partial breast to reduce the risk of local recurrence [2].

In the UK, 40 Gy in 15 fractions is the standard of care for breast radiotherapy [3]. Recently the results of the FAST_Forward trial validated the use of 26 Gy in 5 consecutive fractions for early-stage breast cancer, post breast conservation surgery [4]. These hypofractionated schedules have demonstrated comparable 5-year local tumour control rates, and acute and late normal tissue effects, with the advantage of reduced overall treatment time and patient convenience [4], [5]. The impact of the COVID19 pandemic on radiotherapy services reaffirms the benefits of these fractionation regimes [6]. Hypofractionation requires an additional level of safety due to the higher fractional dose, tighter margins, and steeper dose gradients than conventional fractionation. To ensure precise dose delivery to the target and sparing of the surrounding heart and lung, interfractional and intrafractional uncertainties must be minimised [7].

Tangential whole breast radiotherapy is generally robust to systematic and random setup errors owing to larger margins; however, setup accuracy and target positioning are crucial in volumetric arc therapy (VMAT) and accelerated partial breast irradiation (APBI), due to the greater impact of interfraction and intrafraction discrepancies on the delivered dose [8].

Typically, an anterior and two lateral tattoos are used as the primary reference for initial patient alignment to ensure reproducibility to the scanned position. In some clinics, additional markings are used to indicate the isocentre or field borders [9]. The quality of the delivered treatments is greatly dependent on the ability to position the patient accurately and reproducibly throughout the course of radiotherapy [10].

Image guided radiotherapy (IGRT) is performed prior to treatment delivery, typically using a bone and soft tissue registration to localise the target and correct for interfraction setup deviations [11], [12]. The tumour bed may be demarcated with surgical clips which aid in the delineation of the target volume and may be used for image registration during IGRT. The frequency of IGRT varies per department, ranging from daily, or the first three fractions and thereafter once weekly [13].

### Challenges associated with breast radiotherapy

Tattoo-based setups pose some challenges, namely, mobility of the skin and corresponding variability of the tattoo positions. Radiation therapists often need to manipulate the skin to ensure correct alignment of the lasers to the tattoo markings [14]. Identification of tattoos against other skin markings, misidentification of current tattoos and those from previous irradiation, or ease of visualisation on darker skin tone patients, proves challenging for therapists and increases the probability of setup errors [14], [15], [16].

Today, there is greater awareness around the cosmetic and psychological impact of tattoos. Some patients may be impartial to these however for others, tattoos may serve as a visible permanent reminder of their challenging and sometimes distressing cancer journey [17]. A group of patients who underwent breast radiotherapy with the use of tattoos or skin marks were surveyed in the USA, indicated they were willing to invest additional resources (travel, time, and money) to avoid tattoos/skin marks [18]. These findings are increasingly more pertinent and should be explored deeper, especially in an era where technological advances have enabled suitable alternatives.

More importantly tattoos do not provide postural information of the arm and chin, which can impact reproducibility and ultimately the target positioning [12], [19]. Ultraviolet (UV)-visible tattoos as a replacement for dark ink tattoos has been explored however it requires additional time locate these during patient setup [9], [15], [18].

Breast tissue is highly deformable and image registration (MV or KV) on bony anatomy alone may result in larger interfraction variation and compromised target coverage [16]. Setup reproducibility is often challenging in patients with higher body mass index or larger pendulous breast [20]. Breast tissue is susceptible to contour changes, which could negatively impact the planned dose distribution. SGRT may address these challenges and provide suitable solution for reproducible breast alignment and great confidence with partial breast irradiation [19], [21], [22].

### Surface guided radiotherapy

SGRT is an optical surface tracking system that analyses the patient surface topography and is sensitive to contour and anatomical variations. These discrepancies are more apparent to the radiation therapist than conventional setup methods and provide quantitative information to aid in the clinical decision-making process [16], [23]. In an era of SGRT, the relevance and reliance on tattoos for accurate patient setup may be diminished [24], [25]. Although omitting tattoos seems a minute change, it is a paradigm shift as radiation therapists have relied on these markings for decades. Advances in technology, can facilitate improvements in the patient experience and satisfaction whilst maintaining a high degree of accuracy [19], [24].

SGRT uses non-invasive, non-ionising optical technology to generate a 3D surface of the patient which is matched to a reference surface either from CT or acquired with the SGRT cameras itself. A rigid or deformable surface registration algorithm computes the differences between the live and reference position, providing real-time feedback/displacements in 6 degrees of freedom (6DOF). Surface registration is based on a region of interest (ROI) (potentially user-defined) as opposed to 3 tattoos or skin marks alone. [12], [25], [26].

**Fig. 1:**
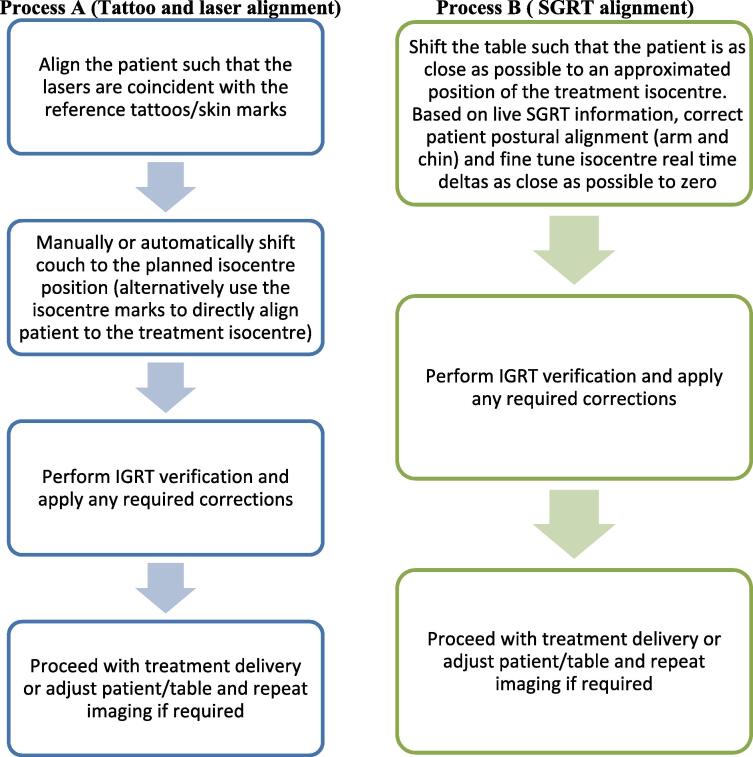
Flowcharts depicting the steps in a typical breast radiotherapy patient setup using conventional tattoos and lasers (process A) vs SGRT (process B).

During traditional tattoo-based setups, the patient is aligned such that the lasers are coincident with the reference marks. The patient is manually or automatically shifted to the planned isocentre position. Alternatively, isocentre marks enables direct alignment to the isocentre (Fig. 1-Process A). The addition of SGRT, allows for fine-tuning of the isocentric alignment in 6DOF including enhanced postural correction. SGRT does not require any visible skin markings. The patient is clinically straightened and shifted directly to the approximated anatomical location. Patient adjustments are based on the real-time displacement information provided by the SGRT system (Fig. 1-Process B). Redundancies in the tattoo/laser-based setup process may improve efficiency [11].

Restricted shoulder mobility and associated discomfort post-surgery may hinder setup reproducibility or result in interfraction or intrafractional discrepancies. The larger field of view (FOV) with SGRT allows for accurate alignment of the patient arm, chin, breast tissue, and surrounding chestwall [11], [16], [27].

Many studies have highlighted SGRT as a convenient and effective tool in improving the accuracy and efficiency of initial patient setup, patient comfort, safety, and clinical outcomes in breast cancer treatments [10], [19], [25]. SGRT is gaining acceptance and adoption and has provided a viable option for tattoo-free patient setup [17], [24], [25]. Where a good correlation exists between the surface and internal anatomy, SGRT offers the potential to reduce the frequency of radiographic imaging and the associated patient imaging dose and may be a suitable alternative on non-imaging days [28], [29], [30]. Real-time non-ionising feedback ensures intrafraction motion can be identified, quantified and promptly controlled. Additionally, radiation delivery can be automatically interrupted if a predefined threshold is exceeded [26].

The aim of this research was to compare the accuracy and reproducibility of SGRT to conventional tattoo-based setups in free-breathing breast radiotherapy and to determine if SGRT can reduce the frequency of routine IGRT. This review focused on SGRT for initial patient positioning in breast radiotherapy compared to conventional tattoo/skin marks and laser-based setups to assess the impact on accuracy, reproducibility and correlation to IGRT.

## Materials and methods

**Fig. 2:**
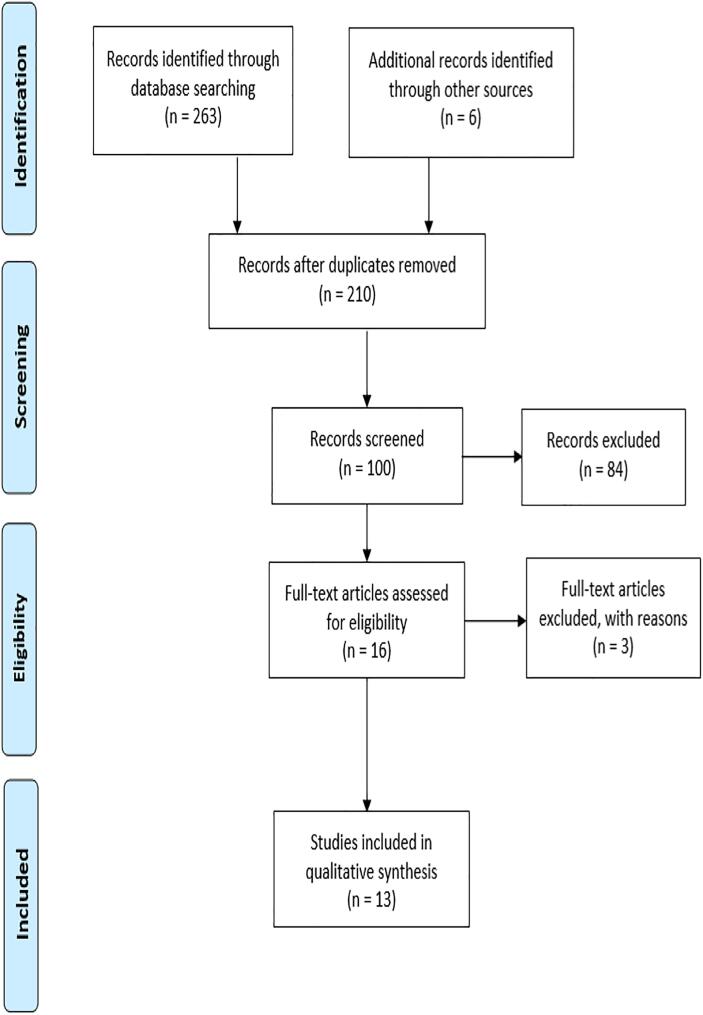
PRISMA flow diagram of information through the phases of the systematic review and corresponding results of the database search, screening and exclusion.

A systematic review of the literature was conducted using the Preferred Reporting Items for Systematic Reviews and Meta-Analyses (PRISMA) [31] (Fig. 2).

Supplementary Table 1 provides the search strategies for all databases. A total of 20 papers were identified in PubMed, 69 in Embase and 42 in Web of Science. A Google scholar search on SGRT and breast radiotherapy limited to 2010–2020 resulted in a further 132 articles.

Breast cancer is most prevalent in females hence this review focused on clinical studies of women receiving breast radiotherapy in free breathing for any disease stage. Left and right-sided whole breast or chestwall were included. All external beam radiotherapy delivery techniques were considered including proton beam irradiation.

Papers needed to report interfractional setup errors based on IGRT analysis and the initial patient setup performed with SGRT and their standard setup procedure (tattoo/skin marks). In studies exploring tattoo-less radiotherapy, SGRT for initial patient alignment was mandatory. Studies analysing intrafraction motion management with SGRT were included if the authors reported setup error data for both setup methods. Phantom studies or those conducted using inhouse developed optical surface tracking systems were excluded. SGRT systems needed to be commercially available with the relevant FDA clearance or CE marking. The full inclusion and exclusion criteria are provided in Supplementary Table 2.

Papers that that met the inclusion criteria, were selected for further screening. These papers were appraised in detail, including their reference list for other relevant articles. The effective public health practice project (EPHPP) quality assessment tool for quantitative studies was used to assess the quality of the literature included in the review and provided an overall rating. Various components were assessed and scored which included study design, data collection and analyses to name a few. A global rating was there decided per paper with a score of 1–3 indicating strong, moderate and weak quality [32]. A summary of the reviewed articles is given in Supplementary Table 3.

Systematic and random translational and rotational setup errors for SGRT and conventional tattoo setups were extracted. Where reported, the mean residual errors and 3D vector displacements were obtained from the included papers. IGRT (MV/KV planar imaging or CBCT) is the current clinical gold standard for patient setup verification. Following initial patient alignment, the acquired radiographic images are registered to the planning reference image and these displacements constitute the residual setup errors. Image registration criteria varies per department however generally based on bone or bone and soft tissue. The group mean systematic and random setup errors are defined by Batin et al [33] as the mean of all the individual mean patient errors and average of standard deviation (SD) of all the individual patients means, respectively.

## Results

This review analysed tattoo and SGRT-based patient positioning in terms of residual setup errors and the correlation of SGRT to IGRT. 16 full papers were assessed and 3 were excluded*.*

The papers reviewed encompassed breast or chestwall treated with tangential technique, locoregionally, or partial breast irradiation with photon or proton therapy. The study population in 10/13 papers was in excess of 20 patients (range: 10–76). However, Stanley et al [25] did not report the number of patients but rather a total of 6000 fractions with 600–900 per tumour site for each setup method.

A summary of the included studies is given in Supplementary Table 3. The imaging modality and registration method used varied between studies as per departmental protocol; however, in nearly 50% of all studies, orthogonal KV imaging was used. Rotational values were not provided in all papers.

Generally, 3 skin markings (range: 2–6) were used as a reference for initial patient alignment. The radiation therapists visually verified that the laser coincided with the anterior and lateral tattoos. For SGRT setups, the displacements based on the surface registration of the current patient position against the DICOM or SGRT reference surface, were reduced as close as possible to zero. The general observed SGRT tolerances were 2–3 mm and 2-3degrees for translations and rotations, respectively.

The overall observations of this review indicate SGRT setups were more accurate compared to the traditional tattoo/laser-based methods with respect to the systematic, random errors and the 3D vector shifts. Mean systematic and random errors in the vertical, longitudinal, and lateral directions were larger for tattoo setups*.* SGRT patient alignment resulted in a consistent reduction in the overall 3D vector shifts.

### Systematic errors

**Fig. 3:**
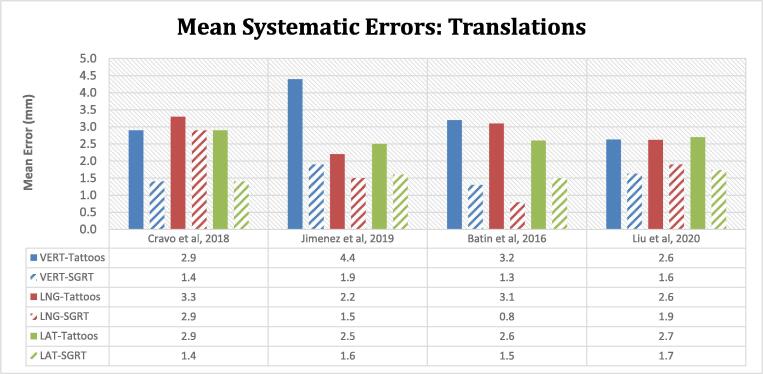
Mean systematic errors are displayed for both setup modalities (tattoo/laser and SGRT). Mean is representative of the mean of all the individual patient mean errors in the 3 translational directions (VERT- vertical, LNG- longitudinal, LAT-lateral). A reduction in the mean systematic errors in the vertical, longitudinal, and lateral directions are seen when SGRT is utilised (indicated by the striped bars). The treatment sites include whole breast, partial breast irradiation, and proton beam chest wall irradiation. Significant improvements are noted in the lateral direction for Cravo et al and all 3 directions for Batin et al.

The SGRT mean systematic errors in the 3 translational directions were reduced when compared to the tattoo setups alone (Fig. 3*)*. The mean systematic translational errors ranged between 2.2 and 4.4 mm for tattoo and laser-based setups and were constrained to within 0.8–2.9 mm with SGRT. In the vertical and lateral directions, average reductions in the residual errors were in the region of 50% (1–2.5 mm) and 40% (0.9–1.5 mm) respectively with SGRT. Greater variability in the percentage difference between both modalities was seen in the longitudinal direction. The systematic rotational errors were comparable for both tattoos and SGRT setups and typically below 1 degree (range: 0.4–1.2°).

### Random errors

**Fig. 4:**
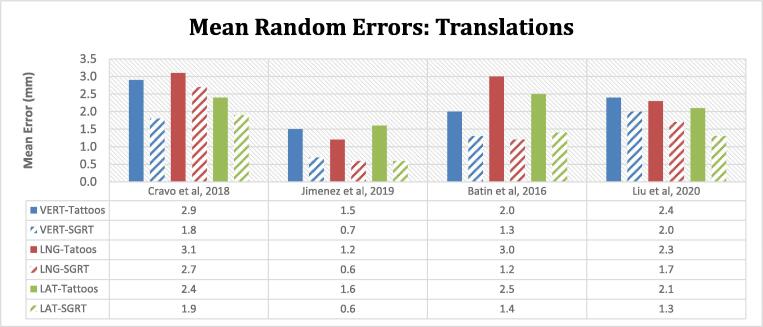
The histogram indicates the mean translational random errors for tattoo (solid) and SGRT setups (striped bars). Random error is defined as the mean or root mean square (RMS) of the standard deviation (SD) of all the individual patients' means. Similar to the mean systematic errors, there is a corresponding reduction in the magnitude of the random errors in all 3 directions when SGRT is used to correct the initial patient position.

Similarly, reductions in the magnitude of the random translational errors were seen in the vertical, longitudinal, and lateral directions with the utilisation of SGRT. The percentage change between both methods ranged between 13 - 63% (0.4–1.8 mm) (Fig. 4*)*. Jimenez et al [24] reported only translational errors. The random rotational errors were comparable for both setup methods (range: 0.6–1.5°).

Substantial improvements were seen by Batin et al [33] for SGRT systematic and random translational and rotational errors. Residual errors with tattoo alignment were reduced from 3.2 ± 2.0 mm, 3.1 ± 3.0 mm, 2.6 ± 2.5 mm in the vertical, longitudinal and lateral directions to 1.3 ± 1.3 mm, 0.8 ± 1.2 mm and 1.5 ± 1.4 mm with SGRT.

### Mean residual setup errors

**Fig. 5:**
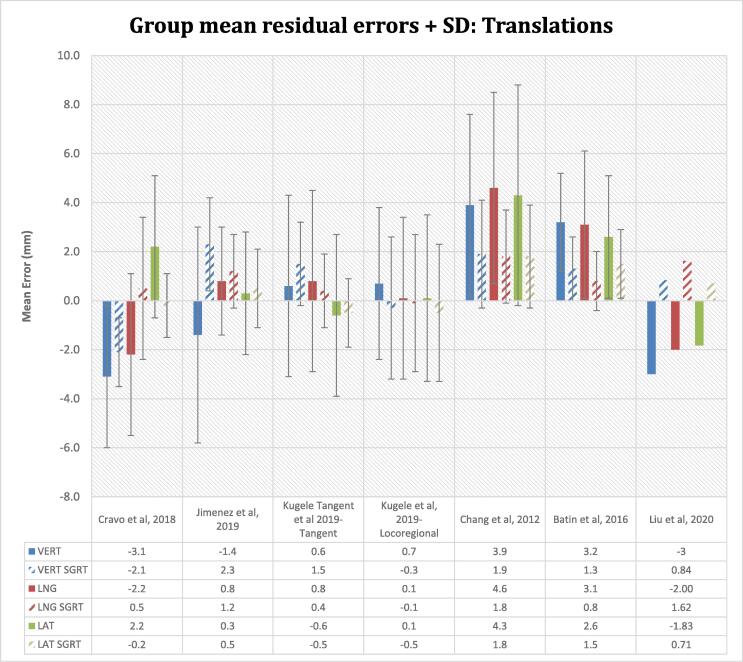
Group mean residual setup errors and its SD for the studies reviewed. The histogram represents the mean of all the mean individual patients’ errors. The SD deviation is indicated by the error bars. SD is not reported by Liu et al. The mean patient positional discrepancies are reduced for SGRT setups however in Jimenez et al and Kügele et al (tangent cohort) an improvement in the vertical direction is seen with tattoo/laser-based setups. The distribution and variability of the shifts are consistently lower for SGRT assisted setup. The methodology and analysis used by Liu et al varies from other studies which explains the directional variations despite a reduction in the mean residual setup errors with SGRT.

The mean positional discrepancies of tattoo-only alignment were larger than with SGRT (Fig. 5 and Supplementary Table 4). However, 2 studies demonstrated a larger vertical error for SGRT setups. Jimenez et al [24] observed a non-statistically significant reduction in systematic and random errors with SGRT however the mean vertical error was larger with SGRT (2.3 vs −1.4 mm). Kügele et al [12] also reported a larger mean vertical error in the SGRT breast tangent field group (1.5 ± 1.7 mm vs 0.6 ± 3.7 mm, p < 0.01). Tattoo setups demonstrated a larger variability in setup errors in all 3 translational directions. The SD with SGRT was consistently smaller than that of the tattoo-only group demonstrating a higher degree of consistency. In some cases, the magnitude of the errors was reduced however the shifts were in opposite directions.

### 3D vector displacements

**Fig. 6:**
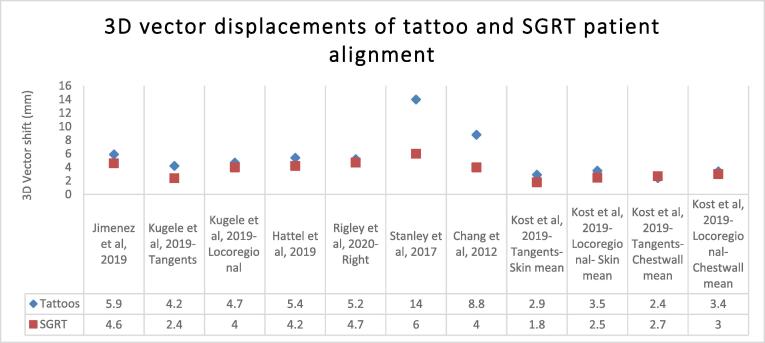
3D vector shifts for free-breathing breast radiotherapy patients. A reduction in the average magnitude of the 3D shift corrections were seen with SGRT setups, suggesting improved setup accuracy. Residual setup errors for tattoos were in the range of 2.4–14 mm and 1.8–6 mm with SGRT. Statistically significant differences were noted for Kügele et al (Tangents and locoregional groups) p < 0.01, Stanley et al (p < 0.01), Chang et al (p = 0.02, <0.05)), Kost et al (Tangents- skin mean) p < 0.001) and in lateral direction for Hattel et al (P = 0.0009).

The 3D vector shifts of the SGRT translational setup errors were consistently smaller than that of the tattoo setups (Fig. 6*)*. Statistically significant differences were noted for Kügele et al [12] (Tangents and locoregional groups respectively) (4.2 vs 2.4 mm and 4.7 vs 4 mm, p < 0.01), Stanley et al [25] (14 ± 7 mm vs 6 ± 2 mm, p < 0.01), Chang et al [22] (8.8 ± 4.2 mm vs 4 ± 2.3 mm, p = 0.02), Kost et al [34] (Tangents and locoregional- skin mean groups respectively) (2.9 ± 1.3 mm vs 1.8 ± 1.0 mm and 3.5 ± 1.9 mm vs 2.5 ± 1.4 mm, p < 0.001). Lateral setup errors reported by Hattel et al [21] were significantly improved (p = 0.0009) with SGRT however the contrary was noted in the vertical direction (p = 0.00004).

Reductions in the magnitude of the SGRT 3D vector shifts were observed by Jimenez et al [24], Rigley et al [17], and Kost et al [34] (tangent and locoregional chestwall mean errors) however these were not statistically significant.

Overall magnitude of the residual setup uncertainties for SGRT and tattoos were in the range of 1.8–6 mm and 2.4–14 mm respectively, with 91% of reported SGRT errors below 5 mm (Fig. 6 and Supplementary Table 5).

### SGRT and IGRT correlation

**Fig. 7:**
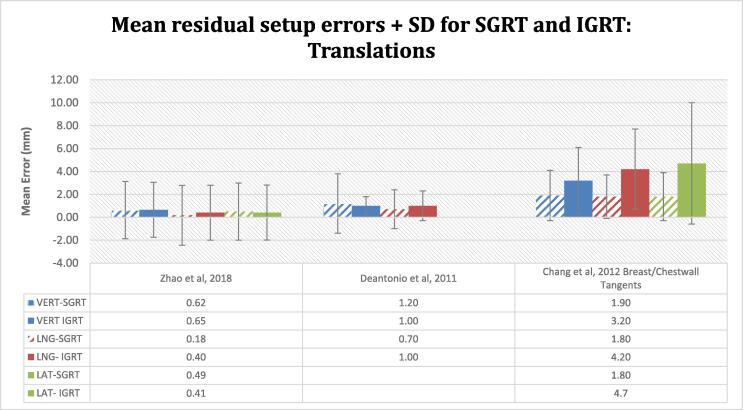
Group mean residual translational errors + SD for SGRT and IGRT. A good correlation of SGRT to IGRT is seen in Ma et al with comparable residual setup errors and SD for both methods. Similarly, in Deantonio et al, a paired *t*-test showed non-significant systematic error in the longitudinal (p = 0.69) and lateral (p = 0.67) directions. Only vertical and longitudinal errors were reported as MV portal images were used for image verification. Chang et al observed improved setup accuracy with SGRT compared to tattoo/laser-based setup supported with a substantial reduction in the residual setup errors. The difference in the vector spatial deviation between both methods is statistically significant for SGRT setups (P < 0.05). A higher degree of variability is seen with tattoo-based setups.

The inclusion of SGRT, improved the setup accuracy compared to tattoo/laser-based setups or was at least of similar magnitude, as verified by IGRT. Chang et al [22] demonstrated, a substantial reduction of the mean residual setup errors in the vertical, longitudinal and lateral directions with SGRT alignment (1.9 ± 2.2, 1.8 ± 1.9, 1.8 ± 2.1 mm) compared to tattoo setups (3.2 ± 2.9, 4.2 ± 3.5, 4.7 ± 5.3 mm) analysed on orthogonal KV imaging (Fig. 7). A corresponding statistically significant reduction in the SGRT 3D vector was seen (4.0 ± 2.3 mm vs 8.3 ± 3.8 mm, p = 0.02). The distribution of the errors with tattoo setups indicates a higher degree of variability. Ma et al [35] suggested a good correlation between SGRT and IGRT with comparable mean setup errors and standard deviations. Similarly, Deantonio et al [36], showed non-significant systematic error in the longitudinal (p = 0.69) and lateral (p = 0.67) directions for both setup methods. An improvement in the vertical random error was noted in favour of IGRT (1.2 ± 0.4 mm vs 1.6 ± 0.6 mm).

### Efficiency

Jimenez et al [24] reported a reduction in the average setup time from 8.0 ± 0.7 to 6.8 ± 0.8mins with SGRT for tattooless breast radiotherapy patient positioning. Similarly, in Ma et al [35] the total time for setup, registration, and correction with CBCT (5.1 ± 0.2 min) is substantially reduced for SGRT alignment (1.1 ± 0.3 min). Batin et al [33] achieved significant time savings in setup and treatment delivery (11 vs 6mins) for tattoo and SGRT setups, respectively. In contrast, Kost et al [34] reported a slight increase in the average SGRT setup time from 5.4 to 6.3 min.

## Discussion

This review aimed to determine the setup accuracy of SGRT compared to conventional tattoo/laser-based setup in breast radiotherapy and its correlation to IGRT (radiographic imaging). IGRT has evolved substantially over the past decade and encompasses various modalities that improve the quality of radiotherapy by minimising geometric uncertainties. SGRT is one such non-ionising surfacing imaging modality [37].

The residual displacements post-IGRT were used for this systematic review. The hypothesis that SGRT is superior to conventional tattoo-based setups, is supported by the observed reductions in systematic, random errors, and the 3D vector displacements. SGRT improved the accuracy and reproducibility of patient setup compared to tattoo setups alone.

### Systematic and random errors

The mean systematic errors in all translational directions were consistently reduced when compared to tattoo alignment. Systematic errors with tattoo setups ranged between 2.2 and 4.4 mm with average reductions of 0.4 to 2.5 mm, with SGRT. The mean systematic errors for SGRT were consistently below 2 mm, except for the longitudinal of 2.9 mm reported by Cravo et al [38]. Similarly, the mean random errors for SGRT were below 2 mm (except 2.7 mm for Cravo et al in the longitudinal direction). The mean longitudinal errors were larger for both tattoos and SGRT groups in the Cravo et al study [38] which could be predominantly attributed to chestwall motion as a result of breathing. Although the vertical and longitudinal mean systematic and random errors are reduced for the SGRT cohort, the impact of breathing motion is still present.

The mean translational random errors indicate a greater degree of variability in the tattoo-only setup groups which ranged between 1.5 and 3.1 mm compared to 0.6–2.7 mm with SGRT. Significant reductions in systematic and random errors were reported by Batin et al [33] in patients undergoing chestwall irradiation with proton therapy, with SGRT-aided initial alignment. The residual setup errors with SGRT were well within the 3 mm robustness for pencil beam scattering.

The mean rotational errors were equivalent for tattoo and SGRT positioning. Traditionally the entire patient alignment is based on a few isolated tattoos/skin markings. However, SGRT provides a larger FOV, a ROI that encompasses the entirety of the treatment area and provides translational and rotational discrepancies. Rotational information can be used to physically manoeuvre and correct patient position prior to IGRT [16]. From the limited rotational error data available, both SGRT and tattoos provided good alignment to within 1degree. Larger reductions in the random rotational errors were seen in Batin et al [33] and reductions of 0.1–0.4degrees across the other studies. Variations in the shape and size of the SGRT ROI between studies could potentially impact the surface registration results. Additionally, differences in setup procedure and IGRT registration methods were present between studies. The evaluated residual rotational errors from IGRT were based around the isocentre accounting for a limited subset of data and not the entire thorax and arm position.

The systematic and random errors for SGRT in this review were maintained in the majority of cases sub 2 mm and 1degree in all translational and rotational directions. These improvements can be attributed to a larger FOV for postural correction and a ROI that incorporates patient topography and the target. During breast radiotherapy patients are typically immobilised on a breastboard or wingboard with one or both arms raised. Good postural alignment in breast radiotherapy is important as positional discrepancies of the arm can lead to larger interfraction setup deviations at the isocentre [12], [16], [9], [39]. Padilla et al [16] observed subsequent longitudinal shifts. Similarly, Kügele et al [12] noted larger setup deviations in the tangential and locoregional patient groups resulting from incorrect placement of the patient arm. Visual feedback provided by SGRT, allows for postural correction (including the arm and chin) prior to IGRT and thereby minimises the residual errors. This cannot be achieved with tattoo/laser-based setups [12]. Variability of tattoo positions due to skin elasticity and the need to manipulate the patient’s skin to match the lasers during the initial alignment, could be a contributing factor to the larger observed residual systematic setup errors. Tattoos are usually placed further away from the target with sole reliance on 2–6 points. The number of tattoos should not significantly influence the observed systematic errors.

### Mean residual setup errors

This review found that the mean residual errors for SGRT (range: −2.1–2.3 mm) were consistently smaller than that of tattoo-only setups (-3.1 – 4.6 mm). There was a corresponding smaller variability in the distribution of the errors indicated by a smaller SD with SGRT. However, the residual errors in the vertical direction in 2 of the studies by Jimenez et al [24] and Kügele et al (Tangent group) [12] appear larger for SGRT compared to tattoo/laser-based setups (2.3 vs −1.4 mm and 1.5 vs 0.6 mm respectively). Although a larger vertical error is observed in the SGRT group, Jimenez et al [24] suggest non-significant differences in the residual errors (5.9 vs 4.6 mm) between both setup methods based on surgical clip match on IGRT. The larger setup errors could be attributed to exacerbated chestwall motion with free-breathing techniques, compared to DIBH [38]. Additionally, the patient is potentially more relaxed at treatment compared to CT which is manifested in the vertical discrepancies and tattoos may be less sensitive to this occurrence [21].

Tattoo/laser-based setup as analysed on portal imaging is less sensitive to detecting soft tissue changes during the course of treatment, resulting from swelling or resolution of seromas, which could also explain this observation. Six studies reported that SGRT was sensitive in detecting errors in the vertical direction with smaller anterior-posterior discrepancies and may be beneficial for heart and lung dose sparing [12], [22], [29], [33], [38], [39]. To account for breathing motion a new SGRT reference surface can be acquired post-IGRT and the user selects a surface that corresponds with the appropriate portion of the respiratory curve (e.g., mid-breathing). Alternatively, the SGRT system may average the patient breathing over a few seconds [12], [36].

### 3D vector displacements

The results of this review indicate that the magnitude of the 3D vector shifts for SGRT was consistently reduced and in cases significantly less than the tattoo cohort, with reductions as large as 57% (14 vs 6 mm). 4 of the reviewed papers reported statistically significant reductions by a magnitude of 0.7 to 8 mm with SGRT compared to tattoos alone. Similarly, residual setup error reductions were reported in an additional 3 papers (range 0.3–1.3 mm) but were not statistically significant.

Breast tissue is a non-rigid structure and challenging to position reproducibly; however, SGRT takes into consideration a larger area of patient surface topography and target, as opposed to just the tattoos, which explains the improved setup accuracy [21], [40]. Equally, variation in the arm position is detected and corrected thus minimising the influence of breast tissue deformation on isocentre accuracy [36]. Similar improvements were seen with the use of SGRT across other clinical applications. Stanley et al [25] observed statistically significant improvements in the 3D vector displacements with SGRT in a large sample of patients for various anatomical sites. Pelvis/lower extremities, abdomen, chest/upper extremities saw reductions in the magnitude of the 3D shift vector from 9 ± 4 mm to 6 ± 3 mm, 10 ± 5 mm to 5 ± 3 mm, and 9 ± 6 mm to 5 ± 3 mm, respectively.

Skin markings are a useful surrogate for initial patient alignment however breast tissue is a superficial target that may not correspond directly to a bony match on the radiographs. MV portal images may still be utilised for verification in breast treatments, despite their limited image contrast, FOV, increased dose and challenge of interpreting the impact of rotations. The reliance on MV and KV imaging alone may result in underestimation of setup errors compared to CBCT and does not account for breast tissue deformation or reproducibility of the arm position [16], [22], [41], [42]. SGRT is sensitive to these discrepancies and provides the user with additional postural and isocentric information [12]. The use of a ROI that encompasses the entire breast and surrounding chestwall improves correlation to IGRT rather than bony anatomy match alone. This has been observed across a variety of treatment techniques [24], [33].

### Conformal radiotherapy techniques

Jimenez et al [24], Chang et al [22], and Hattel et al [21] that included partial breast irradiation, or a subset of these patients, observed improvements in the setup accuracy and reproducibility with SGRT. Surgical clip matching on IGRT is often used as the ground truth for setup verification with these techniques. Whole breast radiotherapy may be more forgiving of small setup errors; however, partial breast irradiation includes the lumpectomy site and a surrounding margin, thus accurate target registration and setup reproducibility are crucial. Jimenez et al [24] reported a non-significant difference in the systematic and random errors between the tattoo and SGRT groups, suggesting that accurate patient setup can be achieved in APBI with SGRT and 2D surgical clip match, in the absence of any visible skin markings. This reinforces the non-reliance on tattoos in APBI under the above-mentioned conditions.

Chang et al [22] observed improved isocentric setup accuracy in partial breast irradiation with the use of SGRT, resulting in a significant reduction in the 3D vector shifts from 8.8 ± 4.2 mm to 4.0 ± 2.3 mm. An early study conducted by Bert et al [43] concluded a reduction in the mean 3D displacements and improved breast tissue congruence with SGRT compared to lasers and portal imaging alone in APBI. Similar results were seen by Gierga et al [44] with good correspondence between target registration error for surgical clip match and SGRT to within 1 mm.

The noted improvements in setup accuracy and reproducibility with SGRT provide greater confidence for tattooless breast radiotherapy, delivery of highly conformal treatments such as VMAT, partial breast irradiation, and more recently hypofractionated regimes [12], [14], [22], [24], [25], [33], [35]. The latest publication by González-Sanchis et al [45] shows SGRT provides more accurate patient positioning and target localisation compared to skin marks, as evaluated on ExacTrac radiographic image registration of at least 3 surgical clips within the tumour bed.

### SGRT and IGRT correlation

Depending on the anatomical site treated, SGRT could potentially reduce the frequency of routine IGRT if there is a good correlation between surface and internal anatomy [12], [26]. SGRT displacements are provided in relation to the treatment isocentre, thus calibration ensures coincidence between the optical and treatment isocentre to within 1 mm.

The results of this review suggest a good correlation between SGRT and IGRT with residual errors of comparable magnitude for both methods or further reduced with SGRT. As seen in Ma et al [35] the systematic errors were non-significant (p > 0.05) for SGRT and CBCT in vertical, longitudinal and lateral directions (0.62 ± 2.54, 0.18 ± 2.61, 0.49 ± 2.54 mm vs 0.65 ± 2.40, 0.40 ± 2.42, 0.41 ± 2.44 mm). Similarly, Deantonio et al [36] reported errors only in vertical and longitudinal axes based on MV portal images and SGRT was non-inferior to tattoos (1.2 ± 2.6, 0.7 ± 1.7 mm vs 1.0 ± 0.8, 1 ± 1.3 mm). Significant improvements in the residual errors with SGRT compared to laser setup and KV orthogonal imaging (8.8 vs 4 mm, p = 0.02) were reported by Chang et al [22]. SGRT has been noted to improve the accuracy and reproducibility of the initial patient positioning compared to tattoos/lasers alone due to the volume of surface data analysed [35], [39].

Significant reductions in the setup deviations were reported by Kügele et al [12] for SGRT based tangential and locoregional breast treatments. The agreement of SGRT based setups to IGRT within the 4 mm clinical tolerance was higher than that of laser-based setups in tangent treatments (95% vs 84%) and (70% vs 54%) for locoregional treatments. Similar improvements in setup reproducibility were observed by Stanley et al (p < 0.01) and Kost et al (p < 0.001) over tattoo/laser-based alignment [25], [34]. Leong et al [14] indicated that KV imaging prior to CBCT in SABR treatments could be excluded in 80% of fractions with a maintained acceptance level of the CBCT registration. Similarly, Heinzerling et al [46] reported that SGRT replaced the pre-CBCT KV imaging and provided setup accuracy within 5 mm without the reliance on skin markings in thorax SABR treatments.

The observed reductions in residual setup errors and incidence of rejected images with SGRT could allow for the minimisation of routine IGRT in breast radiotherapy. Batin et al [33] highlighted residual errors with SGRT were well within the 3 mm and 2degree threshold and could replace orthogonal KV imaging with improved accuracy, efficiency, and reduction in imaging dose. An additional study by Batin et al [47] suggested initial and weekly IGRT is sufficient for patient positioning and monitoring when SGRT is used daily.

Consideration is given to the lessened impact from breathing motion with DIBH however Laaksomaa et al [41] concluded IGRT is required on the 1st fraction and thereafter SGRT can be used for patient setup with a 3 mm isocentric accuracy, allowing a considerable reduction in IGRT. Weekly IGRT may still be required. Haraldsson et al [48] reported daily SGRT patient alignment with weekly MVCT in tomotherapy delivery in H&N, CNS, and thoracic cancers were feasible with the current target margins and a 4 min mean timesaving per fraction for thorax patients. Moser et al [49] reported similar findings that SGRT improved the accuracy of initial patient setup and only intermittent imaging was required after the 1st fraction image verification.

The reduction of residual setup errors with SGRT and its correspondence to IGRT makes it a suitable tool to reduce the frequency of imaging in breast radiotherapy and the corresponding patient imaging dose [45]. In departments where weekly IGRT verification is performed, SGRT facilitates accurate and reproducible daily alignment on non-imaging days [39].

SGRT assumes a strong correlation between the surface and internal targets which is verified by IGRT [29], [33]. Despite the improvement in the systematic and random translational errors, Cravo et al [38] suggest SGRT should be used with another imaging method due to its superficial characteristics. This sentiment is shared by Hattel et al [21] where an improvement in the residual 3D vector displacements was observed for SGRT (5.4 to 4.2 mm), however, this was deemed not sufficient to replace the daily KV imaging.

### Efficiency

SGRT improved setup accuracy as well as improved treatment efficiency, which could be attributed to redundancies in the tattoo/laser-based setups processes, reduced repeat imaging, or the replacement of daily or routine verification imaging [12], [24], [29], [33], [35], [36], [45], [47].

### Limitations of this review

ROI delineation is important for SGRT surface registration and there were variations between the studies in this review. Chestwall patients may have a particularly uniform chest contour and the ROI should include suitable topography for accurate SGRT surface registration [35]. This influence together with breathing motion may exacerbate the variation observed in the longitudinal direction [41], [47]. Breath hold techniques could be beneficial in reducing interfractional and intrafractional variations [38]. Rigley et al [17] showed comparable setup accuracy with right breast but statistically significant improvements for left breast cancers treated with DIBH and SGRT.

The reference surfaces used varied between studies; however, Padilla et al [16] reported no significant differences between DICOM and SGRT reference surfaces. A survey conducted in the USA on the use of SGRT, revealed no distinct benefit of the SGRT over DICOM reference surfaces and vice versa [50]. The DICOM reference is often preferred as it reproduces the initial planned position. In cases where the patient’s anatomy changes over a course of treatment, the SGRT reference surface may be useful.

## Conclusion

For decades, skin marks and tattoos have been utilised in radiotherapy for initial patient alignment. The psychological implications of permanent skin markings are well documented. The evolution of SGRT with modern radiotherapy offers the ability to effect a change in clinical practice by negating the reliance on tattoos for patient setup in breast or chestwall treatments; whilst improving accuracy, patient experience, satisfaction, and quality of life. The results of this review suggest SGRT addresses the challenges associated with conventional tattoo-based alignment and augments the setup procedure providing a more accurate postural and isocentric setup. There appears to be a good correlation between SGRT and IGRT which could reduce the frequency of imaging and the associated patient dose.

The aim of this study was not to compare the setup duration between both methods; however, there is evidence to suggest efficiency gains with SGRT which could positively impact the patient experience, radiotherapy staff, and the department alike. Continuous intrafraction motion management and automatic beam control with SGRT, allows for an additional level of safety with continually advancing radiotherapy delivery techniques.

The trends observed in this review, further support the hypothesis that SGRT improves treatment setup accuracy without the permanent visual reminder of tattoos.

## Declaration of Competing Interest

Wesley Naidoo is employed by VisionRT Ltd however this review was conducted independently with no influence or financial contribution from the employer.
