# Supplementary material for: Feasibility of surface guided radiotherapy for patient positioning in breast radiotherapy versus conventional tattoo-based setups- a systematic review
Source: Tech Innov Patient Support Radiat Oncol. 2022 Apr 16;22:39–49. doi: 10.1016/j.tipsro.2022.03.001 (PMC9035716; doi:10.1016/j.tipsro.2022.03.001)
Supplement: Supplementary data 1 [file mmc1.docx]

|  | **Embase** | **PubMed** | **Web of science** | **Google scholar** |
| --- | --- | --- | --- | --- |
| **Results (Articles)** | 20 | 69 | 42 | 132 |
| **Search Strategy** | (“Surface guided radiotherapy”[Title/Abstract] OR “surface guided radiation therapy”[Title/Abstract] OR SGRT[Title/Abstract] OR “3D surface imaging”[Title/Abstract] OR “surface imaging”[Title/Abstract] OR “optical surface imaging”[Title/Abstract] OR “Surface image guided radiotherapy”[Title/Abstract] OR “surface guided imaging”[Title/Abstract] OR “surface guidance”[Title/Abstract]) AND (“Setup displacement*”[Title/Abstract] OR “setup accuracy”[Title/Abstract] “Set up displacement*”[Title/Abstract] OR “set up accuracy”[Title/Abstract] OR “systematic error*”[Title/Abstract] OR “random error*”[Title/Abstract] OR “setup error*”[Title/Abstract] OR “set up error*”[Title/Abstract] OR “interfractional variation*”[Title/Abstract] OR “set up accuracy”[Title/Abstract] OR “setup accuracy”[Title/Abstract] OR “Set up verif*”[Title/Abstract] OR “setup verif*”[Title/Abstract] OR “set up confirm”[Title/Abstract] OR “setup confirm”[Title/Abstract] OR “displacement verif*”[Title/Abstract] OR “displacement confirm*”[Title/Abstract] OR “residual error*”[Title/Abstract]) AND ("Breast Neoplasms"[Mesh] OR “Breast cancer*”[Title/Abstract] OR “Breast tumor*”[Title/Abstract] OR “breast tumour*”[Title/Abstract] OR “breast neoplasm*”[Title/Abstract] OR “Breast radiotherapy”[Title/Abstract] OR “partial breast irradiation”[Title/Abstract] OR “accelerated partial breast irradiation”[Title/Abstract] OR “breast irradiation”[Title/Abstract] OR “whole breast radiotherapy”[Title/Abstract]) | (('surface guided’ OR ‘Surface image guided’ OR ‘Surface guided imag*’) NEAR/2 (radiotherapy OR ‘radiation therapy' OR 'radiation oncology')):ti,ab  (‘3D surface imag*’ OR ‘surface imag*’ OR ‘optical surface imag*’ OR ‘surface guidance’):ti,ab  #1 OR #2  ((image* OR imaging OR geometric OR ‘set up’ OR setup) NEAR/6 (verif* OR confirm*)):ti,ab  ((offline OR 'off line' OR online OR 'on line' OR ‘set up’ OR setup) NEAR/3 (correction* OR re-planning OR replanning)):ti,ab  (position* NEAR/3 (reproducibil* OR verif* OR accuracy OR accurate OR confirm* OR innaccur*)):ti,ab  ((setup OR 'set up' OR displacement) NEAR/6 (verif* OR confirm* OR accurac* OR error*)):ti,ab  ((systematic OR random OR residual) NEAR/3 (error OR setup OR set-up)):ti,ab  ((Adapt*) NEAR/3 (radiat* OR radiotherapy OR protocol* OR strateg* or correction* OR management)):ti,ab  #4 OR #5 OR #6 OR #7 OR #8 OR #9  'breast radiotherapy'/exp OR 'breast cancer'/exp  (Breast NEAR/3 (cancer OR carcinoma* OR tumo?r* OR neoplasm*)):ti,ab  (Breast NEAR/3 (irradiation OR radiotherapy OR ‘radiation therapy’ OR ‘radiation oncology’)):ti,ab  #11 OR #12 OR #13  #3 AND #10 AND #14 | TS =((((“surface guided” OR “Surface image guided” OR “Surface guided imag*”) NEAR/1 (radiotherapy OR “radiation therapy” OR “radiation oncology”)) OR (“3D surface imag*” OR “surface imag*” OR “optical surface imag*” OR “surface guidance”)) AND (((image* OR imaging OR geometric OR “set up” OR setup) NEAR/5 (verif* OR confirm*)) OR ((offline OR “off line” OR online OR “on line” OR “set up” OR setup) NEAR/2 (correction* OR re-planning OR replanning)) OR (position* NEAR/2 (reproducibil* OR verif* OR accuracy OR accurate OR confirm* OR innaccur*)) OR ((setup OR “set up” OR displacement) NEAR/5 (verif* OR confirm* OR accurac* OR error*)) OR ((systematic OR random OR residual) NEAR/2 (error OR setup OR set-up)) OR ((Adapt*) NEAR/2 (radiat* OR radiotherapy OR protocol* OR strateg* or correction* OR management))) AND ((Breast NEAR/2 (cancer OR carcinoma* OR tumo?r* OR neoplasm*)) OR (Breast NEAR/2 (irradiation OR radiotherapy OR “radiation therapy” OR “radiation oncology”)))) | SGRT and breast radiotherapy |

**Table 1: Search strategies used for each database; Embase, PubMed, Web of Science and Google scholar, and the corresponding number of articles**

**Table 2: Detailed inclusions and exclusion criteria for eligibility for review based on study type, population and reported outcomes/endpoints**

| **Inclusion criteria** | | **Exclusion criteria** |
| --- | --- | --- |
| **Type of studies** | Clinical patient studies  Peer reviewed publications  Retrospective or prospective study design  English language | SGRT accuracy studies performed on phantoms |
| **Participants/Study population** | Females  Supine position  Left or right whole breast, partial breast or chest wall radiotherapy  External beam radiotherapy (3DCRT, IMRT, VMAT)  Proton therapy  Tattoo-less procedure  Tattoos/skin markings (in the comparator group)  Any dose fractionation schedule  Presence of surgical clips or none  Commercially available SGRT systems | Studies conducted using in-house developed optical surface tracking systems |
| **Outcome measures** | *Papers should report the following outcomes/endpoints*:  Translations and/or rotational residual setup errors for both SGRT and tattoo groups as analysed on IGRT (MV/KV portal images or CBCT).  Random and systematic errors, mean errors, 3D vector/root mean square/vector spatial deviation for both setup methods | *Papers reporting the following will not be considered for review:*  Evaluation of breast setup accuracy based on IGRT alone (SGRT not used for initial alignment) |

**Table 3: Summary of the 13 full papers included in this review under 2 main categories in line with the aim and objectives; SGRT vs tattoo and laser-based setups, and SGRT and IGRT correlation**

| **Author and year** | **Title/Description of paper** | **Number of participants** | **Site: Breast or chestwall** | **IGRT modality** | **SGRT tolerances (mm)** | **IGRT tolerances (mm)** | **Study results/outcomes** | **Quality of study- EPHPP rating** |  |
| --- | --- | --- | --- | --- | --- | --- | --- | --- | --- |
|  |  |  |  |  |  |  |  |  |  |
| Shah et al, 2012 | Clinical evaluation of interfractional variations for whole breast radiotherapy using 3-dimensional surface imaging | 50 | Breast | MV portal | Rotations <2degrees | 3mm | SGRT reduced setup errors when compared with patient alignment to skin marks and MV portal imaging. Greater systematic and random errors seen with skin marks alone. | 1 |  |
|  |  |  |  |  |  |  |  |  |  |
| Cravo et al, 2018 | Radiotherapy setup displacements in breast cancer patients: 3D surface imaging experience | 20 | Whole breast | CBCT | Unknown | - | Translational random and systematic errors were higher in the group setup with tattoos alone, compared to SGRT. Although SGRT is beneficial, it should be used with another imaging method. | 1 |  |
|  |  |  |  |  |  |  |  |  |  |
| Jimenez et al, 2019 | Tattoo free setup for partial breast irradiation: A feasibility study | 20 | APBI | 2D Orthogonal (KV) | 3mm, 3degrees | 5mm | SGRT and 2D matching to surgical clips provided excellent accuracy in APBI patient alignment and setup verification with reduced setup time relative to the tattoo cohort. Skin‐based tattoos may no longer be warranted for patients receiving external beam APBI. | 1 |  |
|  |  |  |  |  |  |  |  |  |  |
| Kügele et al, 2019 | Surface guided radiotherapy (SGRT) improves breast cancer patient setup accuracy | 63 | Breast tangents | MV field images | 2mm, 3degrees | - | Laser‐based setup can with advantage be replaced by SGRT based setup. Daily SGRT improved patient setup without additional imaging dose to breast cancer patients. | 1 |  |
|  |  |  |  |  |  |  |  |  |  |
|  |  | 76 | Locoregional breast | Orthogonal KV or MV images |  |  |  |  |  |
|  |  |  |  |  |  |  |  |  |  |
| Hattel et al, 2019 | Evaluation of setup and intrafraction motion for surface guided whole‐breast cancer radiotherapy | 10 | Whole breast and 2 partial breast | Orthogonal KV images | 3mm, 3degrees | - | SGRT showed marked improvements over laser and tattoo setup, however it should not replace the daily orthogonal kV imaging. | 1 |  |
|  |  |  |  |  |  |  |  |  |  |
| Rigley et al, 2020 | Radiotherapy without tattoos: Could this work? | 23 | Right breast or Chest wall | CBCT | 3mm, 3degrees | - | The elimination of tattoos together with SGRT offers a comparable set-up for right sided breast treatments against the traditional tattoo method. | 1 |  |
|  |  |  |  |  |  |  |  |  |  |
| Stanley et al, 2017 | Comparison of initial patient setup accuracy between surface imaging and three-point localization: A retrospective analysis | *600-900 fractions per setup method | Breast | CBCT | - | - | Overall 3D shift corrections for SGRT setup were significantly smaller than with tattoos. SGRT is a viable option for initial patient setup and may be preferable to permanent marks for specific clinics and patients. | 1 |  |
|  |  |  |  |  |  |  |  |  |  |
| Chang et al, 2012 | Video surface image guidance for external beam partial breast irradiation | 23 | Accelerated partial breast irradiation | Orthogonal KV | - | - | SGRT improves setup accuracy to within 2mm compared to orthogonal KV imaging or lasers. Potential to reduce PTV margins for APBI. | 1 |  |
|  |  |  |  |  |  |  |  |  |  |
| Kost et al, 2019 | Assessment of Setup Accuracy Using Anatomical Landmarks for Breast and Chest Wall Irradiation with Surface Guided Radiation Therapy | 32 non DIBH | Tangents- Skin mean | MV Portal | - | - | Based on anatomical landmark analysis, SGRT improved patient setup accuracy with a slight, but clinically nonsignificant increase in setup time. | 1 |  |
|  |  |  |  |  |  |  |  |  |  |
|  |  |  | Locoregional- Skin mean |  | - |  |  |  |  |
|  |  |  |  |  |  |  |  |  |  |
|  |  | 30 non DIBH | Tangents- Chest wall mean |  | - |  |  |  |  |
|  |  |  |  |  |  |  |  |  |  |
|  |  |  | Locoregional- Chest wall mean |  | - |  |  |  |  |
|  |  |  |  |  |  |  |  |  |  |
| Batin et al, 2016 | Can surface imaging improve the patient setup for proton postmastectomy chestwall irradiation? | 15 | Chest wall | Orthogonal KV | 3mm, 2degrees | 2mm, 1degree | SGRT allows postmastectomy chest wall patients to be positioned more accurately for proton irradiation and substantially more efficiently than radiograph-only based techniques. | 1 |  |
|  |  |  |  |  |  |  |  |  |  |
| Liu et al, 2020 | Application of Optical Laser 3D surface imaging system (Sentinel) in breast cancer radiotherapy | 49 | Breast | CBCT | - | - | SGRT reduced the residual setup errors in all 6 directions and can be applied in positioning for breast cancer patient accurately without unnecessary imaging dose. | 1 |  |
|  |  |  |  |  |  |  |  |  |  |
| **SGRT and IGRT Correlation** | | | | | | | | |  |
| Ma et al, 2018 | Optical Surface Management System for Patient Positioning in Interfractional Breast Cancer Radiotherapy | 20 | Breast | CBCT (translations only) | 5mm (translations) | - | SGRT is an efficient tool to improve the accuracy and increase the speed for verifying the patient positioning in radiotherapy for breast cancer. | 1 |  |
|  |  |  |  |  |  |  |  |  |  |
| Deantonio et al, 2011 | Detection of setup uncertainties with 3D surface registration system for conformal radiotherapy of breast cancer | 15 | Breast | MV portal | - | - | SGRT is fast, simple, non-invasive and seems to be reliable in detecting patient setup errors and could be used to assess the setup reproducibility for breast cancer patients. | 1 |  |
|  |  |  |  |  |  |  |  |  |  |
| Chang et al, 2012 | Video surface image guidance for external beam partial breast irradiation | 23 | Accelerated partial breast irradiation | Orthogonal KV | - | - | SGRT is a more accurate method for isocentre placement in comparison to conventional laser-based alignment or orthogonal kilovoltage imaging of the chest wall. | 1 |  |
|  |  |  |  |  |  |  |  |  |  |

**Table 4: Group mean residual setup errors and their standard deviations for the reviewed studies**

| **Author and year** | **Modality** | **Mean residual errors (+SD): Translations (mm)** | | | | | |
| --- | --- | --- | --- | --- | --- | --- | --- |
|  |  | **VERT (AP)** | **SD** | **LNG (CC)** | **SD** | **LAT (LR)** | **SD** |
| Cravo et al, 2018 | **Tattoo** | -3.1 | 2.9 | -2.2 | 3.3 | 2.2 | 2.9 |
|  | **SGRT** | -2.1 | 1.4 | 0.5 | 2.9 | -0.2 | 1.3 |
| Jimenez et al, 2019 | **Tattoo** | -1.4 | 4.4 | 0.8 | 2.2 | 0.3 | 2.5 |
|  | **SGRT** | 2.3 | 1.9 | 1.2 | 1.5 | 0.5 | 1.6 |
| Kugüle et al, 2019-Tangent | **Tattoo** | 0.6 | 3.7 | 0.8 | 3.7 | −0.6 | 3.3 |
|  | **SGRT** | 1.5 | 1.7 | 0.4 | 1.5 | −0.5 | 1.4 |
| Kügele et al, 2019- Locoregional | **Tattoo** | 0.7 | 3.1 | 0.1 | 3.3 | 0.1 | 3.4 |
|  | **SGRT** | -0.3 | 2.9 | −0.1 | 2.8 | −0.5 | 2.8 |
| Chang et al, 2012 | **Tattoo** | 3.9 | 3.7 | 4.6 | 3.9 | 4.3 | 4.5 |
|  | **SGRT** | 1.9 | 2.2 | 1.8 | 1.9 | 1.8 | 2.1 |
| Batin et al, 2016 | **Tattoo** | 3.2 | 2.0 | 3.1 | 3.0 | 2.6 | 2.5 |
|  | **SGRT** | 1.3 | 1.3 | 0.8 | 1.2 | 1.5 | 1.4 |
| Liu et al, 2020 | **Tattoo** | -3 |  | -2.00 |  | -1.83 |  |
|  | **SGRT** | 0.84 |  | 1.62 |  | 0.71 |  |

**Table 5: 3D vector shifts for tattoo and SGRT-based patient setups in breast radiotherapy within the included papers. The statistical test and significance are also provided**

| **Author and year** | **Modality** | **3D vector shift: Translations (mm)** | **Percentage difference between Tattoos and SGRT** | **Statistical test** | **Statistical significance** |  |
| --- | --- | --- | --- | --- | --- | --- |
|  |  |  |  |  |  |  |
| Jimenez et al, 2019 | Tattoo | 5.9 | 22 | Mann–Whitney or Brown–Forsythe tests | Non-significant |  |
|  | SGRT | 4.6 |  |  |  |  |
| Kügele et al, 2019 | Tattoo- Tangents | 4.2 | 43 | Students t-test for 2 independent mean for the translations and Wilcoxon sum rank test for the vector offset. | SGRT decreased the setup deviation significantly compared to laser-based setup for both tangential and locoregional treatment (P < 0.01) |  |
|  | SGRT- Tangents | 2.4 |  |  |  |  |
|  | Tattoo- Locoregional | 4.7 | 15 |  |  |  |
|  | SGRT- Locoregional | 4 |  |  |  |  |
| Hattel et al, 2019 | Tattoo | 5.4 | 22 | Wilcoxon rank‐sum test with a significance level of 5% | Lateral with SGRT was significantly better (P = 0.0009). Longitudinal and rotation non-significant (P = 0.96 and P = 0.46). Significant improvement in the vertical with tattoo setup (p=0.000004) |  |
|  | SGRT | 4.2 |  |  |  |  |
| Rigley et al, 2020, Right | Tattoo | 5.2 | 10 | T-Test: Two-sample assuming unequal variances | Non-significant (p=0.04) |  |
|  | SGRT | 4.7 |  |  |  |  |
| Stanley et al, 2017 | Tattoo | 14 | 57 | Wilcoxon signed rank test with significance level of <0.01 | Statistically significant (P < 0.01) |  |
|  | SGRT | 6 |  |  |  |  |
| Chang et al, 2012 | Tattoo | 8.8 | 55 | 2-sample t tests. Significance level P<0.05 | Statistically significant (p=0.02) |  |
|  | SGRT | 4 |  |  |  |  |
| Kost et al, 2019 | Tattoo- Tangents Skin | 2.9 ± 1.3 | 38 | Test not stated | Statistically significant (P < 0.001) |  |
|  | SGRT- Tangents Skin | 1.8 ± 1.0 |  |  |  |  |
|  | Tattoo- Locoregional Skin | 3.5 ± 1.9 | 29 |  | Statistically significant (P < .001) |  |
|  | SGRT- Locoregional Skin | 2.5 ± 1.4 |  |  |  |  |
|  | Tattoo- Tangents Chestwall | 2.4 ± 1.9 | 11 |  | Non-significant (p=0.30) |  |
|  | SGRT- Tangents Chestwall | 2.7 ± 1.6 |  |  |  |  |
|  | Tattoo- Locoregional Chestwall | 3.4 ± 2.4 | 12 |  | Non-significant (p=0.15) |  |
|  | SGRT- Locoregional Chestwall | 3.0 ± 2.1 |  |  |  |  |
